# A novel Monte Carlo (MC) dose model for small MLC fields of the cyberknife^®^ M6^TM^ radiosurgery system using the EGSnrc

**DOI:** 10.1002/acm2.13880

**Published:** 2023-01-18

**Authors:** Taindra Neupane, Panagiota Galanakou, Charles Shang, Theodora Leventouri, Michael Kasper, Wazir Muhammad

**Affiliations:** ^1^ Department of Physics Florida Atlantic University Boca Raton Florida USA; ^2^ South Florida Proton Therapy Institute Delray Beach Florida USA; ^3^ Lynn Cancer Institute Boca Raton Regional Hospital Baptist Health South Boca Raton Florida USA

**Keywords:** cyberknife system, Monte Carlo simulations, patient‐specific quality assurance, small field dosimetry

## Abstract

The multi‐leaf collimator (MLC)‐equipped CyberKnife^®^ M6 radiosurgery system (CKM6) (Accuray Inc., Sunnyvale, CA) has been increasingly employed for stereotactic radiosurgery (SRS) to treat relatively small lesions. However, achieving an accurate dose distribution in such cases is usually challenging due to the combination of numerous small fields ≤ (30 × 30) mm^2^. In this study, we developed a new Monte Carlo (MC) dose model for the CKM6 system using the EGSnrc to investigate dose variations in the small fields. The dose model was verified for the static MLC fields ranging from (53.8 × 53.9) to (7.6 × 7.7) mm^2^ at 800 mm source to axis distance in a water phantom, based on the computed doses of Accuray Precision^®^ (Accuray Inc.) treatment planning system (TPS). We achieved a statistical uncertainty of ≤4% by simulating 30–50 million incident particles/histories. Then, the treatment plans were created for the same fields in the TPS, and the corresponding measurements were performed with MapCHECK2 (Sun Nuclear Corporation), a standard device for patient‐specific quality assurance (PSQA). Results of the MC simulations, TPS, and MapCHECK2 measurements were inter‐compared. An overall difference in dosimetric parameters such as profiles, tissue maximum ratio (TMR), and output factors (OF) between the MC simulations and the TPS results was found ≤3% for (53.8 × 53.9–15.4 × 15.4) mm^2^ MLC fields, and it rose to 4.5% for the smallest (7.6 mm × 7.7 mm) MLC field. The MapCHECK2 results showed a deviation ranging from –1.5% to + 4.5% compared to the TPS results, whereas the deviation was within ±2.5% compared with the MC results. Overall, our MC dose model for the CKM6 system showed better agreement with measurements and it could serve as a secondary dose verification tool for the patient‐specific QA in small fields.

## 1 INTRODUCTION

In radiotherapy, accurate dose calculation is crucial as incorrectly calculated dose distributions may lead to detrimental effects on the patients,^1^, ^2^ especially in treatment procedures involving small fields (≤30 mm × 30 mm for a 6MV photon beam), such as stereotactic radiosurgery (SRS), stereotactic body radiotherapy (SBRT) and intensity modulated radiation therapy (IMRT).^3^, ^4^ These treatments contain numerous non‐coplanar, smaller segments/beamlets that add challenges regarding the optimal selection of the detector and evaluation of its response and lead to non‐equilibrium conditions^5^ that contribute to additional dosimetric uncertainties compared with the conventional radiotherapy measurements. Therefore, recommendations and standard guidelines for the use of various detectors and machine‐specific corrections focused on small‐field dosimetry are needed.^6^, ^7^ Indeed, the International Atomic Energy Agency (IAEA) and the American Association of Physicists in Medicine (AAPM) have jointly published international codes of practice (CoPs) and provided new guidelines for relative dosimetry in non‐standard and small fields.^8^, ^9^ According to these CoPs, sensitivity factors are added to a plethora of detectors to translate the dosimetry of the standard fields to the small fields.

In radiation therapy, Monte Carlo (MC) simulations are the most recommended statistical method for accurate dose calculations.^10^, ^11^, ^12^ Comprehensive guidelines are provided by the AAPM Task Group (TG) report such as TG‐105^11^ and TG‐15.^13^ Further, several studies demonstrated MC simulated correction factors for various detectors used in small field dosimetry including diodes, micro‐chambers, point scintillators, and micro‐diamond detectors.^14^, ^15^, ^16^, ^17^, ^18^

The InCiseTM 2 Multi‐Leaf Collimator (IMLC) equipped CyberKnifeM6 radiosurgery system (CKM6) (Accuray Inc., Sunnyvale, CA) is commonly used in the SS/SBRT cancer treatments with flattening filter‐free (FFF) photon beamlets.^19^ The CKM6 can deliver numerous non‐coplanar, small treatment beams with sub‐millimeter accuracy. Its capability of highly precise dose delivery in 6° of freedom and real‐time tumor tracking further improves the efficiency of such treatments.^20^ However, the CyberKnife system is a specialized linac that cannot generate the reference field (i.e., 10 cm × 10 cm at 100 cm source to surface distance SSD) used for conventional dosimetry. Therefore, a hypothetical reference field, called a machine‐specific reference field such as a fixed cone of 60 mm diameter at 800 mm source to axis distance (SAD), was introduced.^7^, ^21^ The IAEA TRS‐483 provides the calibration guidelines for absorbed dose to water traceable to a primary standard laboratory using a reference ion chamber. The comprehensive guidelines, for machine commissioning^19^, ^20^ and periodic quality assurances (QAs)^3^, ^22^ of using CyberKnife for advanced treatments such as SRS/SBRT have been provided in the literature. Appropriate corrections are integrated into the TPS to account for the uncertainties associated with the different field geometries, beam quality, and ion chamber response from reference fields to the machine‐specific reference field.^9^

The MC dose calculation algorithm was first added to the TPS (Accuray Precision^®^, version 1.1) of the CKM6 as an optional tool that could be used for stand‐alone dose calculation or cross‐validation.^23^, ^25^ Even if it could be stand‐alone for dose calculations, due to lack of published data that compares the FSPB and MC algorithms, in this study, we used MC only for cross‐validation. Nevertheless, its dosimetric evaluation was reported later in the literature.^24^, ^25^, ^26^, ^27^ Furthermore, some independent MC dose models to calculate specific correction factors^17^ and to perform patient‐specific QA using the EGS++ class library were also reported.^28^, ^29^, ^30^

The CyberKnife treatment plan requires a consistent and reliable PSQA that should include monitor units (MU) calculation, plan transfer, and delivery, prior to the treatment.^23^, ^24^ A diode array, MapCheck2 (Sun Nuclear Corporation, Melbourne, FL) is a common and reliable detector used in PSQA.^31^, ^32^, ^33^ However, appropriate corrections are needed^33^, ^34^, ^35^ due to its position/orientation, field size dependence, and dose‐response, specifically in small fields. The planning accuracy with small MLC fields was evaluated with possible corrections.^17^, ^24^ Particularly, the treatment plans containing very small fields, less or equal to 15 mm × 15 mm, require greater attention during the dose calculations and measurements.^36^ To deal with such cases, the implementation of proper correction factors during PSQA is crucial.^23^, ^28^

This study aims to: (1) evaluate the feasibility and efficacy of a novel MC dose model for the CKM6 system using general purpose code EGSnrc MC package in which the Incise2 MLC (IMLC) module is designed uniquely to match the manufacturer design, and (2) investigate its potential role in PSQA for the plans consisting of small fields. To the best of our knowledge, this is the first independent MC dose model reported for PSQA application of the CKM6 system whose feasibility and clinical effectiveness have been explored via MapCHECK2 to minimize dose discrepancies. Furthermore, the model is designed to be user‐friendly since it can be performed using the graphical user interface (GUI).

## 2 MATERIALS AND METHODS

### 2.1 Monte Carlo simulations

**FIGURE 1:**
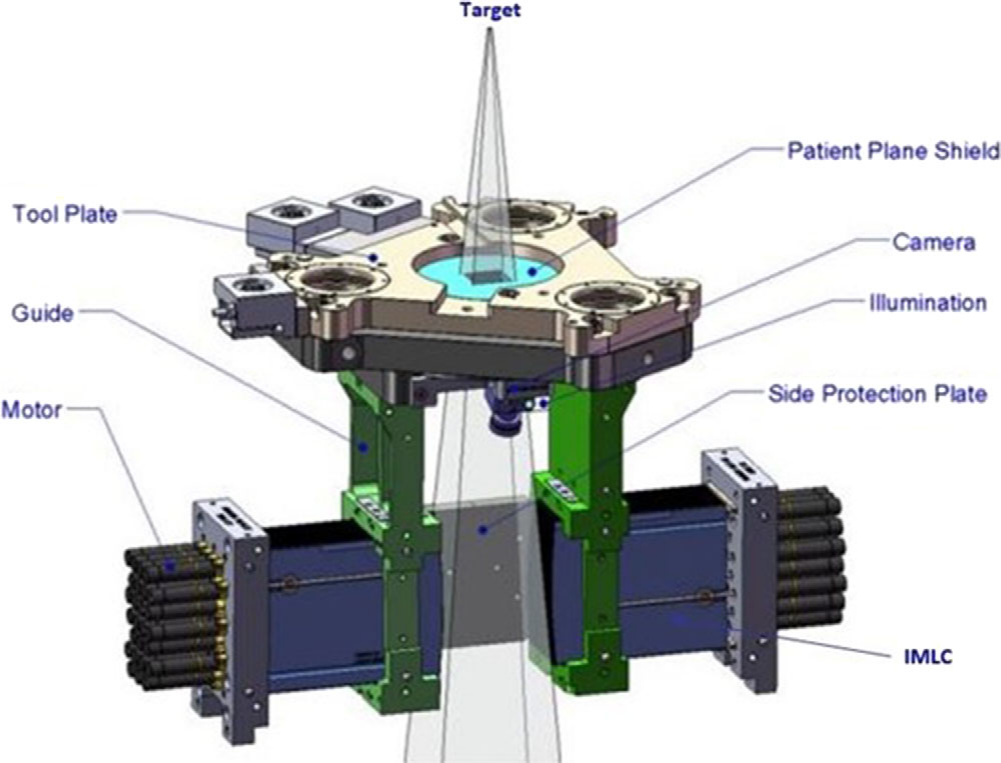
The MLC‐based CyberKnife^®^ (CKM6) linac (Accuray Inc., Sunnyvale, CA) equipped with the Incise™ 2 MLC (IMLC).^15^ The major components of the CKM6 linac head are labeled in the diagram (Adapted from Asmerom et al., 2016)

**FIGURE 2:**
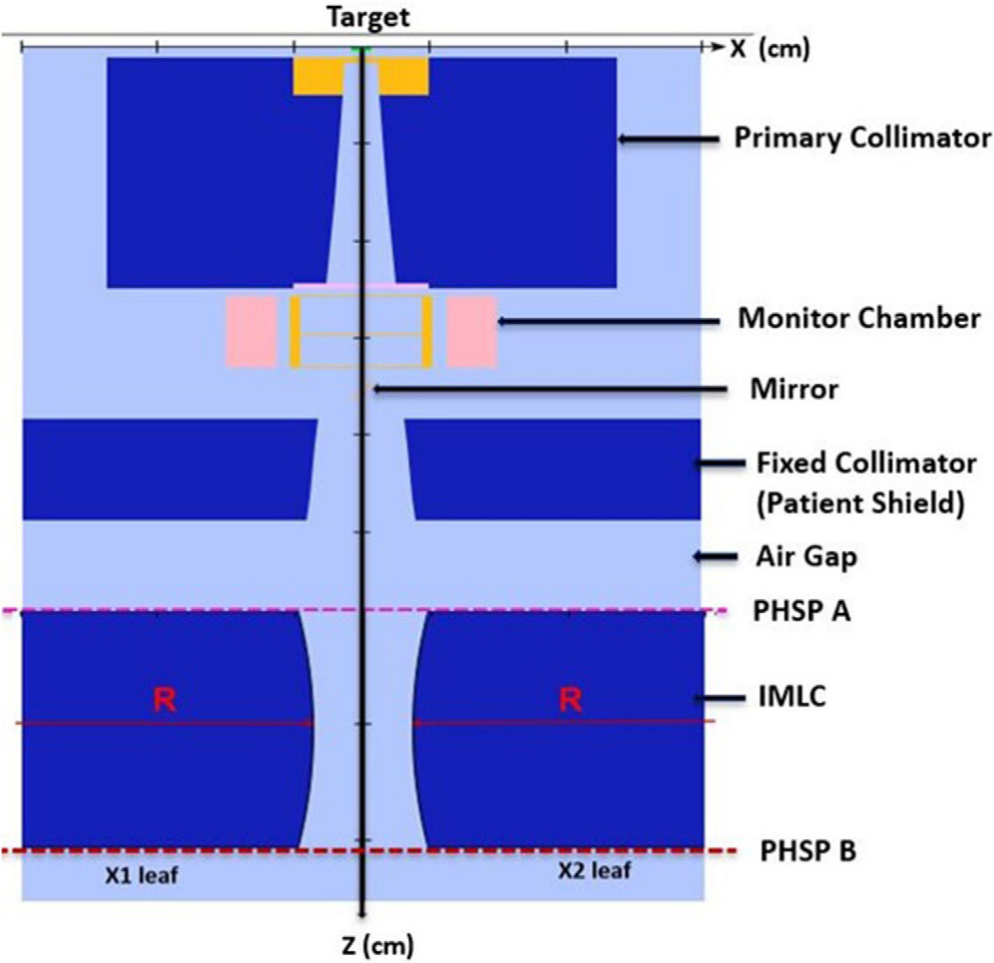
A cross‐sectional view of the MC simulated linac head for the MLC‐based CyberKnife^®^ (CKM6) system using the BEAMnrc program. The PHSP A and PHSP B represent the phase‐space scoring planes defined just above and below the IMLC component respectively during MC simulations. Actual dimensions of the linac components are not displayed due to the non‐disclosure agreement

The EGSnrc Monte Carlo simulation package was used to simulate the IMLC‐based CyberKnife^®^M6 radiosurgery system (CKM6).^19^ The IMLC is used to collimate the radiation beam (i.e., 6 MV flattening filter‐free (FFF) photon) in such a way that the beam can be delivered with numerous non‐coplanar, smaller beamlets from multiple treatment angles with sub‐millimeter accuracy. Figure 1 shows a schematic of the main features/components of the CKM6 head.^19^ The MC simulations were performed in two steps: (1) BEAM modeling of the CKM6 head with or without IMLC using BEAMnrc^37^ and (2) 3D voxelized dose simulations in the phantom DOSXYZnrc.^38^ The beam model (Figure 2) was created by modifying the existing component modules inside the BEAMnrc based on manufacturing details. Those details were provided by the vendor (Accuray Inc.) with a non‐disclosure agreement. The modules such as SLABS (target), CONESTAK (primary collimator), CHAMBER (monitor chamber), MIRROR (mirror), CONESTAK (patient shield), SLABS (air gap), and MLCE (IMLC) were used to design the virtual CKM6 head.

The IMLC module was constructed by modifying the MLCE module (BEAMnrc) to adapt to the actual manufacturing design (Figure 1).^19^ It consists of 26 pairs of tungsten‐alloy (density of 17.75 g/cc) leaves. Each leaf has a width of 3.85 mm at 800 mm source‐to‐axis distance (SAD). A leaf tilt of 0.5° was also applied in the module to minimize inter‐leaf leakage. Round‐shaped leaves (radius of curvature, R) were defined to mimic the actual leaf design. The radius of curvature, R_rad_ ≈ R – 1.2 mm was applied to account for photons’ attenuation from the edges of the leaves. A separate geometrical cross‐section data file was generated using the EGSnrc graphical user interface (GUI) based on data provided by the vendor (Accuray Inc.).

The opening of the IMLC leaves was defined at the mid‐plane (355 mm down from the target) as shown in Figure 2. According to this geometry, the transformation of X co‐ordinates between treatment plans and MC plans were given by^39^:

(1) $$X_{1\;}\;\left(MC\;plan\right)=\;-\left(R_{rad\;}-\frac{SCD}{SAD}*X_{1\;}\left(Treatment\;plan\right)\right)$$

(2) $$X_{2}\;\left(MC\;plan\right)=\left(R_{rad}+\;\frac{SCD}{SAD}*X_{2}\left(Treatment\;plan\right)\right)\;$$

Where, SCD = 355 mm is the source to center (mid‐plane) of IMLC distance, SAD = 800 mm is the source to axis distance, and leaf opening defining a field size,$\;X\;=\;X_{2}-X_{1}$.

MC simulations were performed with or without IMLC for static fields (53.8 × 53.9–7.6 × 7.7) mm^2^ at 800 mm SAD. During the simulation, various parameters (i.e., incident particle/histories [20–50 million] energy [6–7 MeV] and full‐width half maximum [1.8–2.4 mm] of the incident [electron] source and voxel size [1–5 mm] in water phantom) were varied to match the simulation results with the measurements. Various variance reduction techniques such as range rejection, directional bremsstrahlung splitting, and particle splitting were applied to optimize simulation efficiency and reduce the statistical (relative dose) uncertainty.^40^, ^41^ The MC simulation for each field was continued until uncertainty ≤3% was achieved, except for the smallest MLC field (7.6 mm × 7.7 mm), which was ∼4.0%.^11^

Phase‐space file registration is an efficient way to optimize the computational efficiency of MC simulations.^42^ For our simulations, the files were stored at two planes (i.e., PHSP A and PSHP B) above and below the IMLC (Figure 2) to increase efficiency.^37^ Particles in the phase‐space files generated by the BEAMnrc were sampled up to three times specifically for the small fields. The resampling (up to five times) will not induce any systematic bias as the EGSnrc utilizes the history‐by‐history method to determine statistical uncertainties.^41^ Rather, these are based on the number of independent simulated histories.

The MC dose simulations were performed using these phase‐space files on two iso‐centric (at 800 mm SAD) settings: (1) at SSD = 785 mm, z = 15 mm, which mimics a water phantom measurement, and (2) at SSD = 730 mm, z = 70 mm that corresponds with the MapCHECK2 QA setting. The dose‐scoring phantoms were defined as three‐dimensional (3D) water phantoms with sizes of (30 × 30 × 30) cm3 and (20 × 20 × 20) cm^3^. MC simulations were initially performed using a Mac laptop (2.5 GHz Intel Core I5, 8GB RAM) and later with high‐performance computing. The dose outputs (.3ddose files) were extracted and analyzed using MATLAB (The MathWorks.com) software 2018 for the MC model validation.

For the MC simulations, five IMRT treatment plans (step‐shoot technique) were acquired retrospectively and customized (each plan contains five small fields). The plan parameters were extracted (BEAMnrc) from DICOM data (rtplan.dcm) and feed into the DOSXYZnrc for the dose calculations in the phantom. The synchronized MC simulation process involves the BEAMnrc (synchronized module, phase space file before MLC, input file, and sequence file) and DOSXYZnrc (beam shared library, input file). The final output (.3ddose and egsphant) files were extracted using MATLAB and the isodose (95%, 80%, 60%, 50%, and 25%) distributions were compared against the TPS results.

### 2.2 MC model validation

Two‐dimensional (2D) dose profiles obtained from the MC simulations were compared with those obtained from the TPS for static MLC fields (53.8 × 53.9–7.6 × 7.7) mm^2^ at 800 mm SAD. Additionally, 2D dose profiles obtained from commissioning water measurements and by MapCHECK2 PSQA are also compared for MLC fields (53.8 × 53.9–7.6 × 7.7) mm^2^ at 800 mm SAD. The dose profile comparisons were done by determining the full‐width half maximum (FWHM) of each dose profile. Similarly, the dosimetric parameters such as TMR, and OFs were compared to validate our MC model. For any clinical field (*f_clin_*), the TMR, and OF are given by^43^, ^44^

(3) $$TMR\;\left(z,f_{clin}\right)=\;\;\frac{D\left(z,\;f_{clin}\right)}{D\;(z_{max},\;\;f_{clin})}$$

(4) $$OF_{z_{ref}}\;\left(f_{clin}\right)=\;\;\frac{D_{\;}\left(z_{ref},\;f_{clin}\right)}{D_{ref}\;\left(z_{ref},\;f_{ref}\right)}$$

Where *z_max_* and *z_ref are_* the depths of maximum and reference doses, respectively whereas *f_clin_* and *f_ref represent_* clinical and reference fields. The reference field of the 60 mm (diameter) cone, which is equivalent to the 53.8 mm × 53.9 mm MLC field, defined at 800 mm SAD, and the reference depth of 15 mm was used. The TMR values between the MC simulations and water phantom measurements performed during the commissioning of the CKM6 were compared for all the listed static MLC fields. The OF values were computed from the MC simulations, they were also compared with the measurements acquired with the ion chamber (PTW 31014), and diodes (SN Edge and SN MapCHECK2). Additionally, the dose profiles acquired from the MapCHECK2, the MC simulations and TPS (validated with the MapCHECK2 measurements) were evaluated by using 3%/3 mm and 2%/2 mm gamma criteria as per AAPM recommendations.^45^

### 2.3 Retrospective measurements

The treatment plans of 50 SRS/SBRT clinical cases (extra/intra‐cranial) were selected and re‐measured using the MapCHECK2 at 800 mm SAD with 5 cm solid water on the top (build‐up) and 5 cm solid water on the bottom (backscatter) as described by Keeling et al.^32^ The plans selected consisted of various small, IMRT fields/segments at different beam angles/projections such as anterior‐posterior (AP), right‐left (RL) or lateral, and superior‐inferior (SI). A total of 16 out of 50 treatment plans are composed of very small (≤10 mm width) fields/segments. These plans were re‐measured by applying ‐ 4% output correction to correct the dose over‐response of a diode. Such correction was found during the retrospective measurements. A quantitative comparison of measured versus calculated dose maps was conducted within the SNC patient software (version 6.2.0). The change in dose profiles and gamma passing rate with/without applying the “calculate shift” or measurement uncertainty function were also analyzed.^35^ The use of the measurement uncertainty function is to optimize the comparison for better alignment. The gamma passing rates along with gamma criteria were recorded for all measurements and the results were evaluated by separating into two groups: small (width > 10 mm) and very small (width ≤ 10 mm) fields.

### 2.4 Output factors measurements

The verification plans were generated for 10 static MLC fields ranging from 100.0 mm × 100.1 mm to 7.6 mm × 7.7 mm defined at 800 mm SAD using the Accuray Precision^®^ (Accuray Inc., Sunnyvale, CA) TPS. The TPS offers the (FSPB and MC dose calculation algorithms.^25^ Initially, both FSPB and MC algorithms were used to create the verification plans. However, the MC dose calculation required a notably longer calculation time to achieve acceptable uncertainty (≤3%) compared to the FSPB algorithm. Thus, FSPB was used for dose calculations.^26^, ^27^

The corresponding dose/output measurements were performed using a micro‐chamber (PTW 31014), Edge detector (SN), and diode array (SN MapCHECK2). For the ion chamber, and Edge‐detector, the measurement geometry consisted of an iso‐centric setup with 800 mm SAD, and the detectors were placed in a water phantom at 15 mm depth. The MapCHECK2, which has an inherent buildup of 2.0 g/cm2 solid water, was placed at 20 mm depth in a solid water phantom.^31^ For each measurement, 100 MU was delivered, the average reading was recorded after three trials, and then converted to respective doses. The dose recorded for each clinical field (*f_clin_*) was normalized to the machine‐specific reference (*f_msr_*) (i.e., 60 mm cone defined at 800 mm SAD that is equivalent to 53.8 mm × 53.9 mm MLC field at same SAD) for the respective set of measurements.^17^, ^20^ In other words, for broad fields (>30 × 30 mm^2^), the detector‐specific output factor (OF) or outputs ratio (OR) was calculated as^4^, ^46^, ^47^:

(5) $$OF_{det}^{f_{clin}}=OR_{det}^{f_{clin}}\;=\;\frac{M_{Q_{clin}}^{f_{clin}}}{M_{Q_{msr}}^{f_{msr}}}\;\approx \frac{D_{Q_{clin}}^{f_{clin}}}{D_{Q_{msr}}^{f_{msr}}}$$

Where “*det*” represents a detector, the parameters *D*, *M*, and *Q* represent absorbed dose, detector reading, and beam quality factor respectively.

However, for small fields (≤30 × 30 mm^2^), it is calculated as^7^, ^8^, ^9^:

(6) $$\Omega_{Q_{clin}\;Q_{msr}\;}^{f_{clin}\;f_{msr}}=\;\frac{D_{w,\;\;Q_{clin}}^{f_{clin}}}{D_{w,\;Q_{msr}}^{f_{msr}}}\;=\;\frac{M_{Q_{clin}}^{f_{clin}}}{M_{Q_{msr}}^{f_{msr}}}\;*\;k_{Q_{clin},\;\;Q_{msr}}^{f_{clin},\;f_{msr}}$$

Where $\Omega_{Q_{clin},\;\;Q_{msr}}^{f_{clin},\;f_{msr}}$ is the field output factor for the *f_clin_* field with respect to *f_msr_* field and $k_{Q_{clin},\;\;Q_{msr}}^{f_{clin},\;f_{msr}}$ is a correction factor that depends on beam quality (Q), field size (f), detector type, and machine (focal spot).

The measured output factors/ratios for both the MapCHECK2 and Edge detector were compared with the commissioning data incorporated into the TPS^19^, ^20^ for those MLC fields mentioned above. The possible output correction factors were examined specifically for smaller fields and their clinical relevance in PSQA using the MapCHECK2 was studied. In detail, the outputs for 5 × 5 cm, 4 × 4 cm, and 3 × 3 cm were obtained using a PTW Semiflex 31014 ionization chamber (0.015 cc) by measurements against the output of 10 × 10 cm at 95 cm SAD in water (5 cm depth). We then replaced the detector with a SNC Edge diode and PTW 60017 diode for extended measurements down to 0.75 × 0.76 cm. The data were compared with the measurements by Exradin A16 chamber (0.007 cc) in solid water. Per the references, including a table from Indra J Das's presentation—like Figure 9 of AAPM TG 155, we finalized the outputs for the commissioning. MapCheck2 checks were calibrated accordingly for small fields.

## 3 RESULTS AND DISCUSSION

### 3.1 MC modeling and validation

The optimal model (source) parameters to generate the 6 MV FFF clinical beam were E = 7.0 MeV Gaussian electron beam with FWHM = 2.2 mm and 50 million incident particles/histories in simulations. The MC results (dose profiles, TMR, OFs) were found within a 2% difference compared to the TPS results for the equivalent reference MLC field (53.8 × 53.9 mm^2^) defined at z = 15 mm, SAD = 800 mm in a water phantom. The corresponding dose (relative) uncertainty in the MC simulation was ∼2%. However, the dose uncertainty was increased up to 4.0% for the smaller fields, as expected.^11^

**FIGURE 3:**
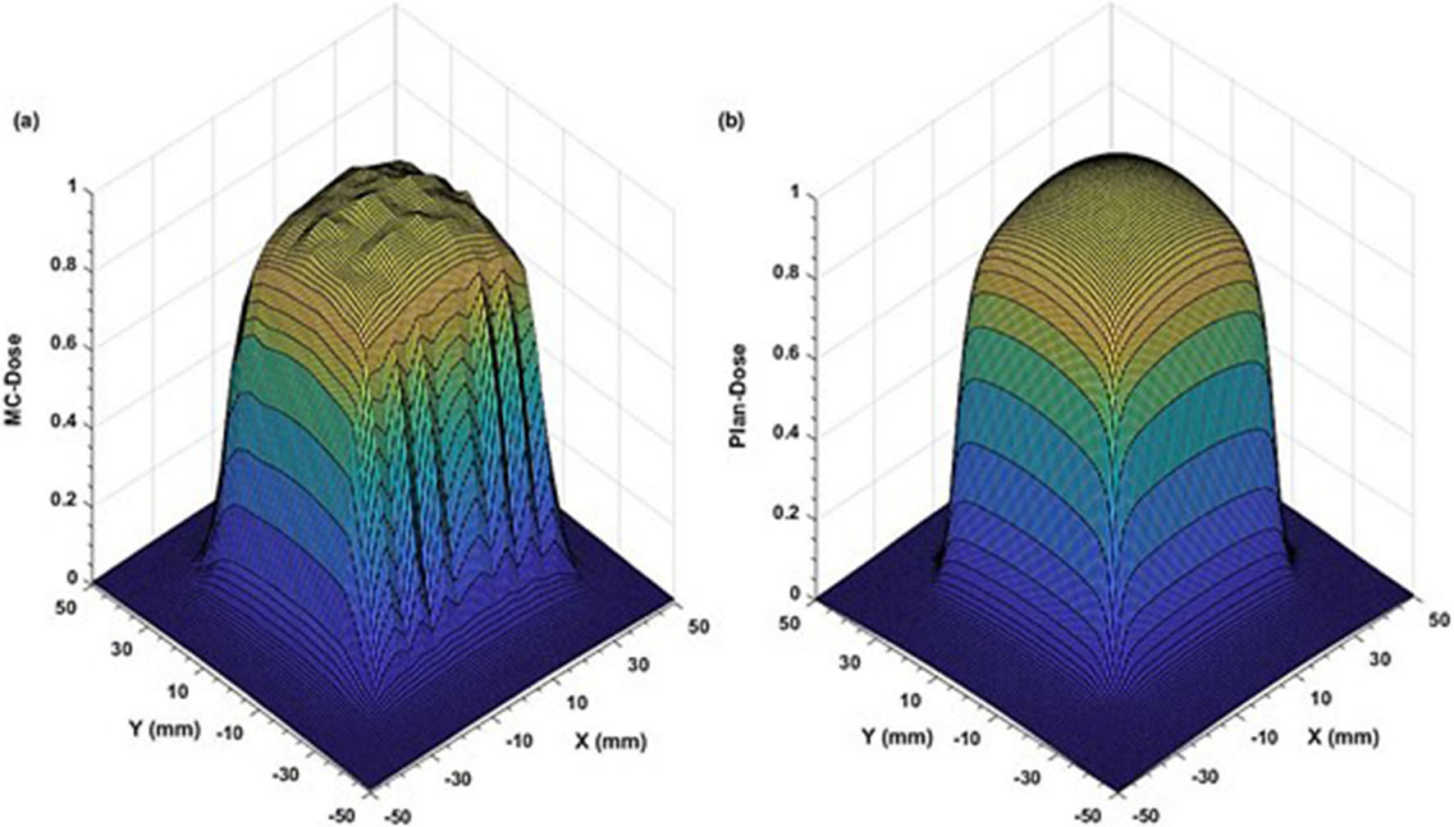
Comparison of two‐dimensional dose profiles obtained from (a) MC simulation and (b) TPS using the MapCHECK QA setup (SSD = 730 mm, z = 70 mm) for the reference MLC field of 53.8 mm × 53.9 mm with 1 mm voxel size in a water phantom

**FIGURE 4:**
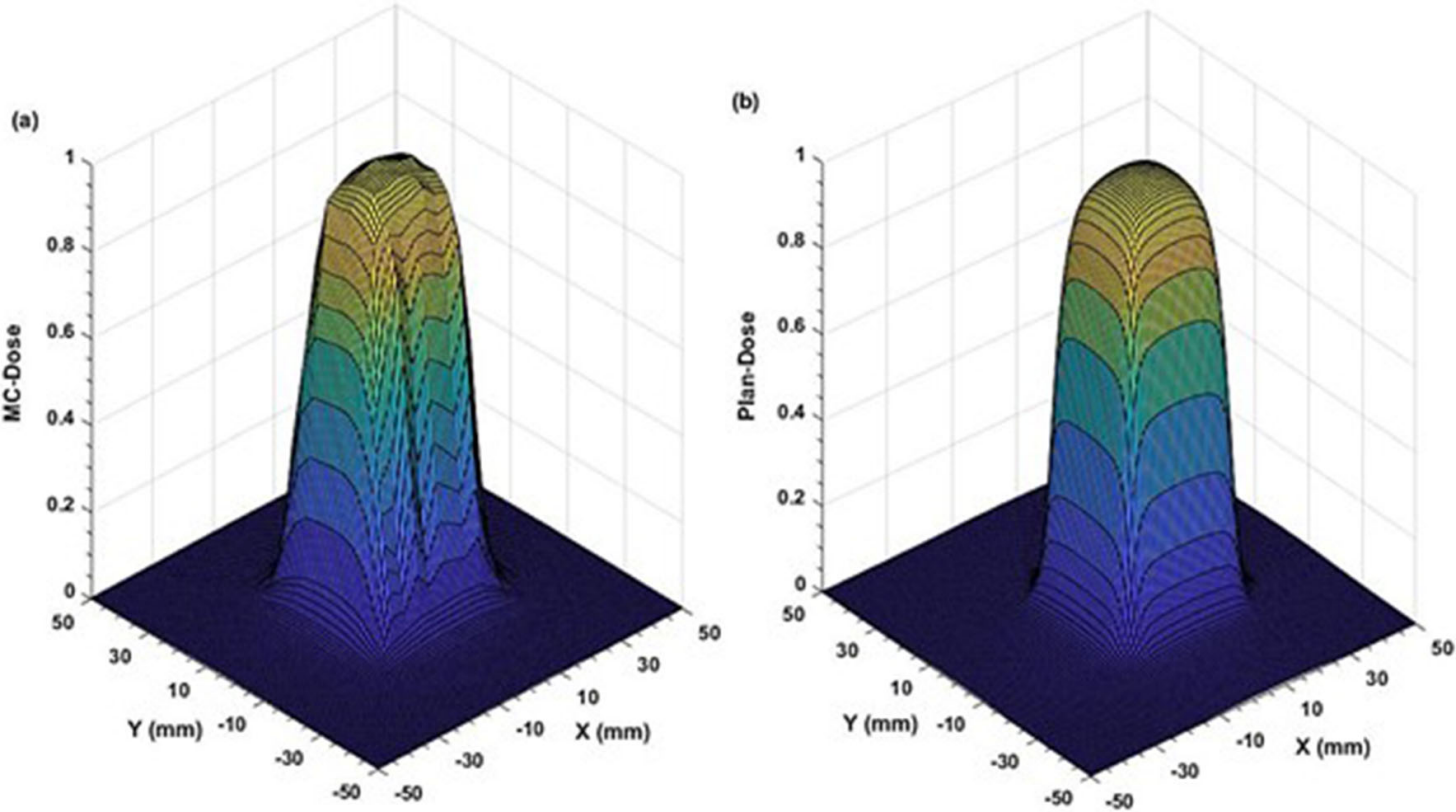
Comparison of two‐dimensional dose profiles obtained from (a) MC simulation and (b) TPS using the MapCHECK QA setup (SSD = 730 mm, z = 70 mm) for 30.8 mm × 30.8 mm MLC field with 1 mm voxel size in a water phantom

**FIGURE 5:**
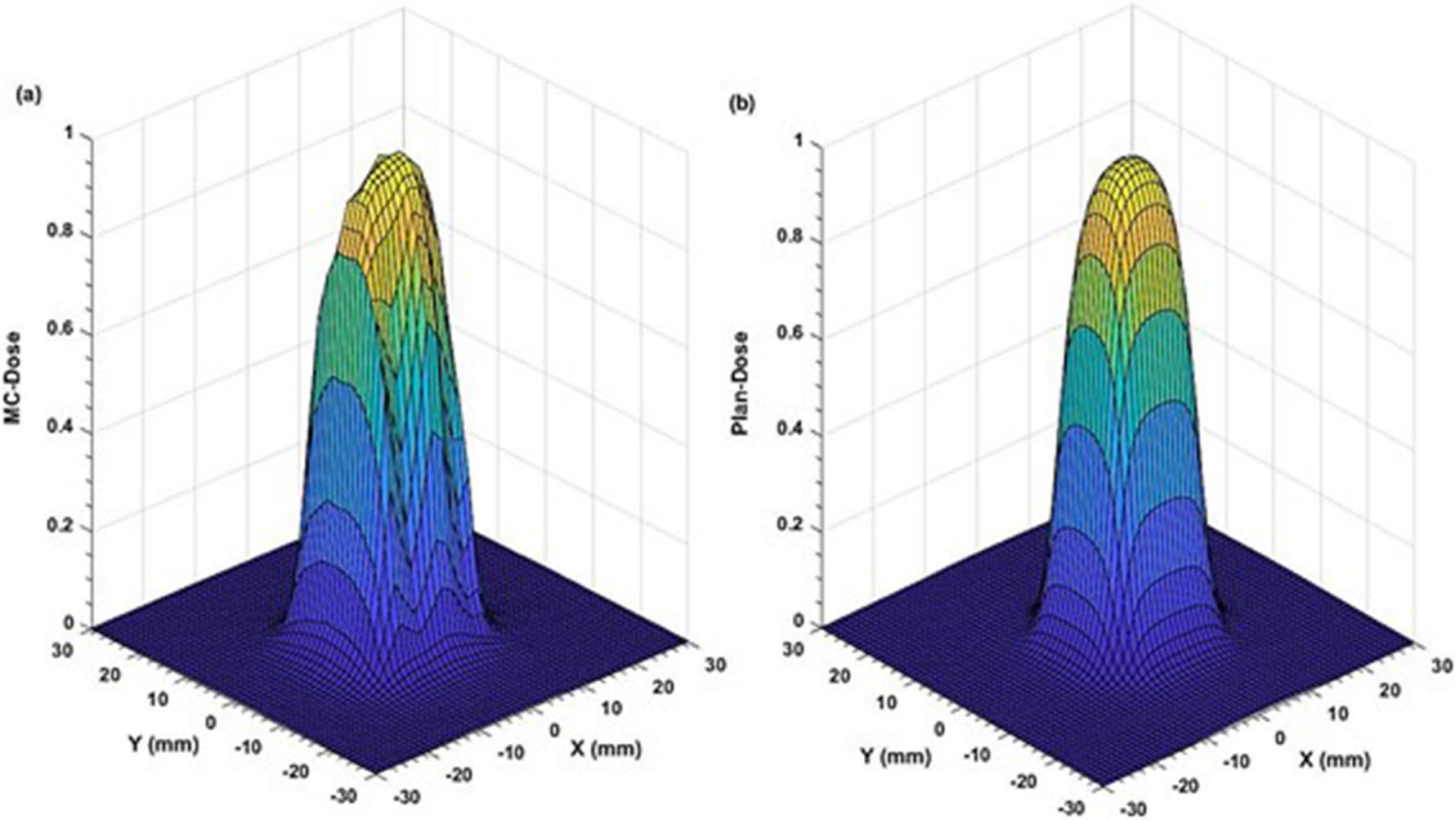
Comparison of two‐dimensional dose profiles obtained from (a) MC simulation and (b) TPS using the MapCHECK QA setup (SSD = 730 mm, z = 70 mm) for 15.4 mm × 15.4 mm MLC field with 1 mm voxel size in a water phantom

**FIGURE 6:**
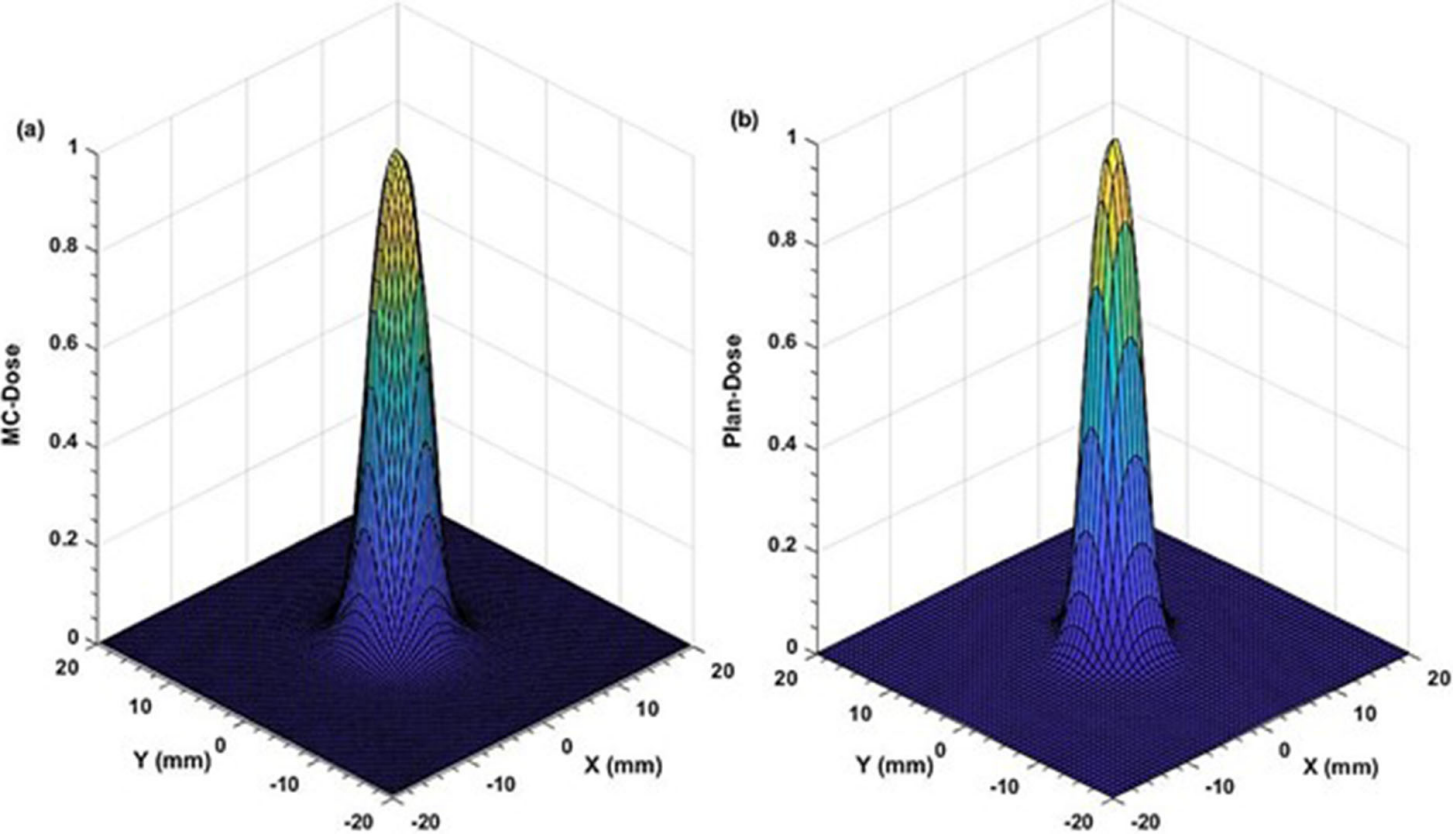
Comparison of two‐dimensional dose profiles obtained from (a) MC simulation and (b) TPS using the MapCHECK QA setup (SSD = 730 mm, z = 70 mm) for the smallest MLC field of 7.6 mm × 7.7 mm with 1 mm voxel size in a water phantom

Comparison of the two‐dimensional (2D) dose profiles based on FWHM calculations, showed ≤2% and ≤3% differences for the (53.8 × 53.9–38.4 × 38.5) mm^2^ and (30.8 × 30.8 – 23.0 × 23.1) mm^2^ fields, respectively (Figures 3 and 4). However, for the two smallest fields (15.4 mm × 15.4 mm and 7.6 mm × 7.7 mm), the differences increased to 4.0 % (Figures 5 and 6). The field size defined by the FWHM of the simulated dose profile was observed slightly wider than the nominal field size defined by the collimator setting (apparent widening effect).^8^, ^46^ This widening effect is more pronounced (up to 0.7 mm or 9% wider) for very small fields as shown in Figures 5 and 6.^4^, ^36^

Apart from well‐matching dose profiles, the MC simulations exhibited more realistic dose distributions (Figures 3, 4, 5, 6) by considering important geometrical features of the IMLC leaves (i.e., width, shape, edge, tip, tilt),^19^, ^20^ and by simulating all particle interactions with the medium transversed. These features of leaves contribute to photons attenuation, scatter, and leakage effects altering the dose distributions. Such contributions are visible in the MC simulated profiles by displaying multiple spikes on the dose‐scoring plane, and uneven sides. On the other hand, the TPS (Figures 3, 4, 5, 6) generates the dose profiles by applying several approximations, smoothing functions, and robust calculation techniques resulting in smoother profiles.^26^, ^27^ Regarding dose output, they remain unaffected for large fields. However, the robustness of dose calculation algorithms can significantly perturb the outputs for the small fields.^48^ These perturbations are typically coming from small‐field related conditions, such as loss of charged particle equilibrium, partial source occlusion, and mismatch of field and detector sizes.^4^, ^46^

**FIGURE 7:**
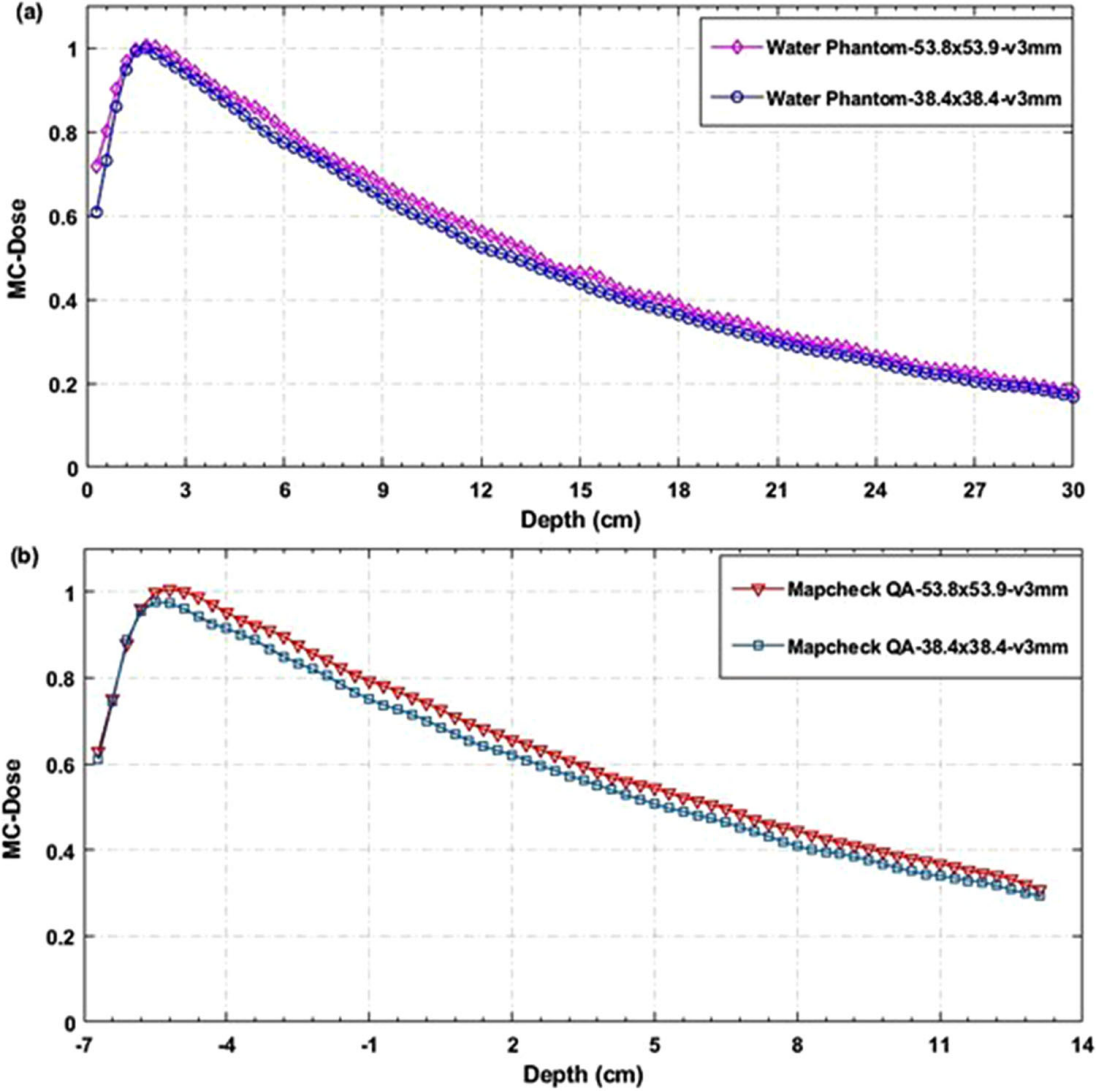
Comparison of MC depth dose curves for the MLC fields (53.8 × 53.9 and 38.4 × 38.4) mm^2^ with 3 mm voxel size between (a) water phantom and (b) MapCHECK QA settings

**FIGURE 8:**
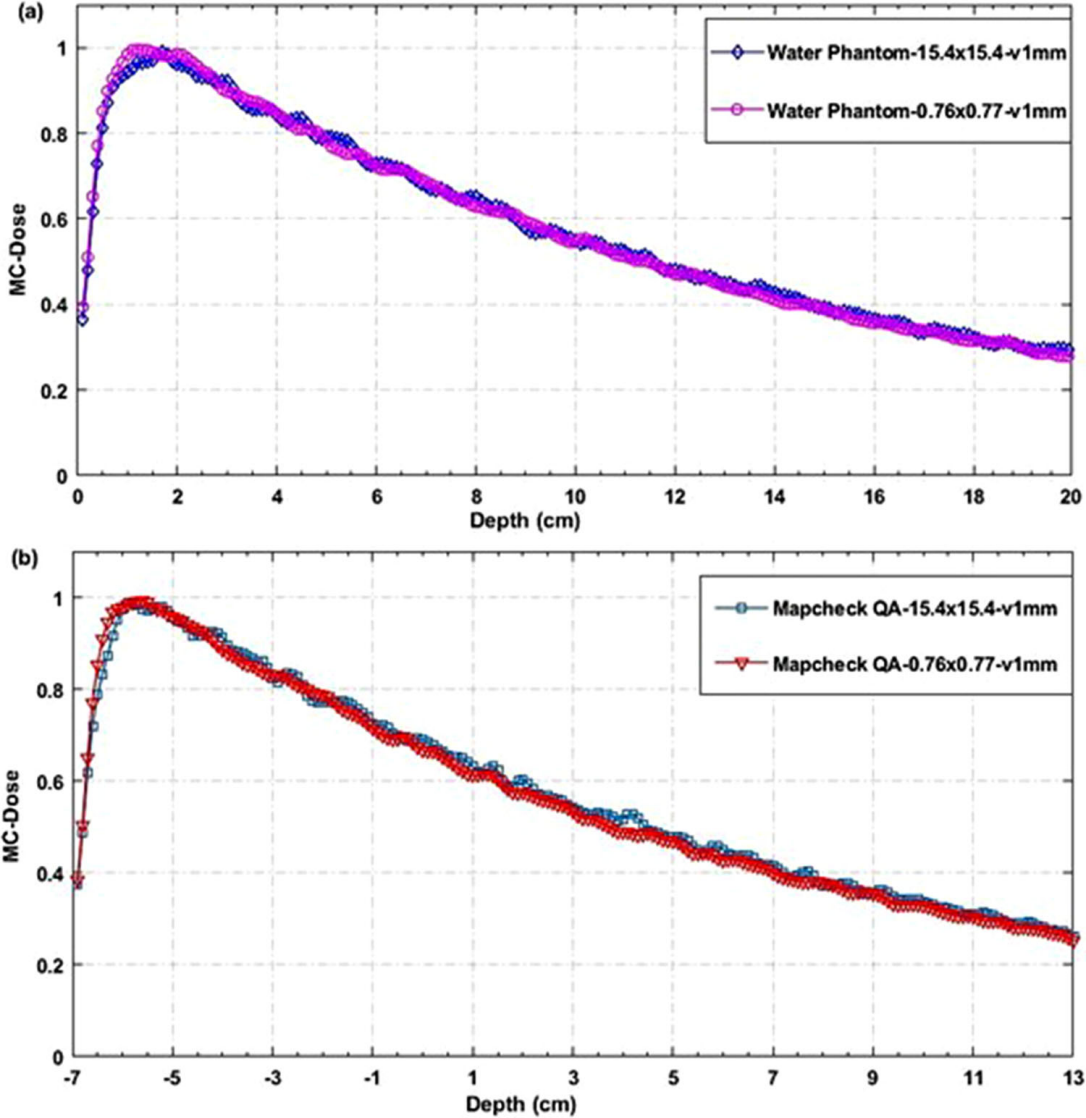
Comparison of MC depth dose curves for the smallest MLC fields (15.4 × 15.4 and 7.6 × 7.7) mm^2^ with voxel size 1 mm between (a) water phantom and (b) MapCHECK QA settings

Figure 7 displays the comparison of MC simulated depth dose curves between two simulation settings for two larger fields (53.8 × 53.9 and 38.4 × 38.4) mm^2^ with 3 mm voxel size. The comparison of MC simulated depth dose curves with the commissioning data by using the TMR method showed a ≤3% difference, which is acceptable based on the AAPM TG‐105 and TG‐157 guidelines. It is noted that the depth dose curves have smooth dose fall‐off along the central axis, and the smoothness of the dose distribution depends on the phantom geometry, composition, and voxel size.^11^ Figure 8 shows the corresponding depth dose curves for two very small fields (15.4 × 15.4 and 7.6 × 7.7) mm^2^ with 1 mm voxel size. The statistical dose uncertainties in MC simulations were 2%–4% from large to small field sizes, while the highest dose uncertainty resulted in the greatest noise in dose distribution. As a result, the depth dose curves for smaller fields seem noisier than the larger fields (Figures 7 and 8). The effect of voxel size from 1–5 mm on the dosimetric accuracy, statistical uncertainty, and simulation efficiency are also examined since an appropriate selection of scoring voxel size is necessary to minimize the dose uncertainty as well as the volume averaging effect.

**FIGURE 9:**
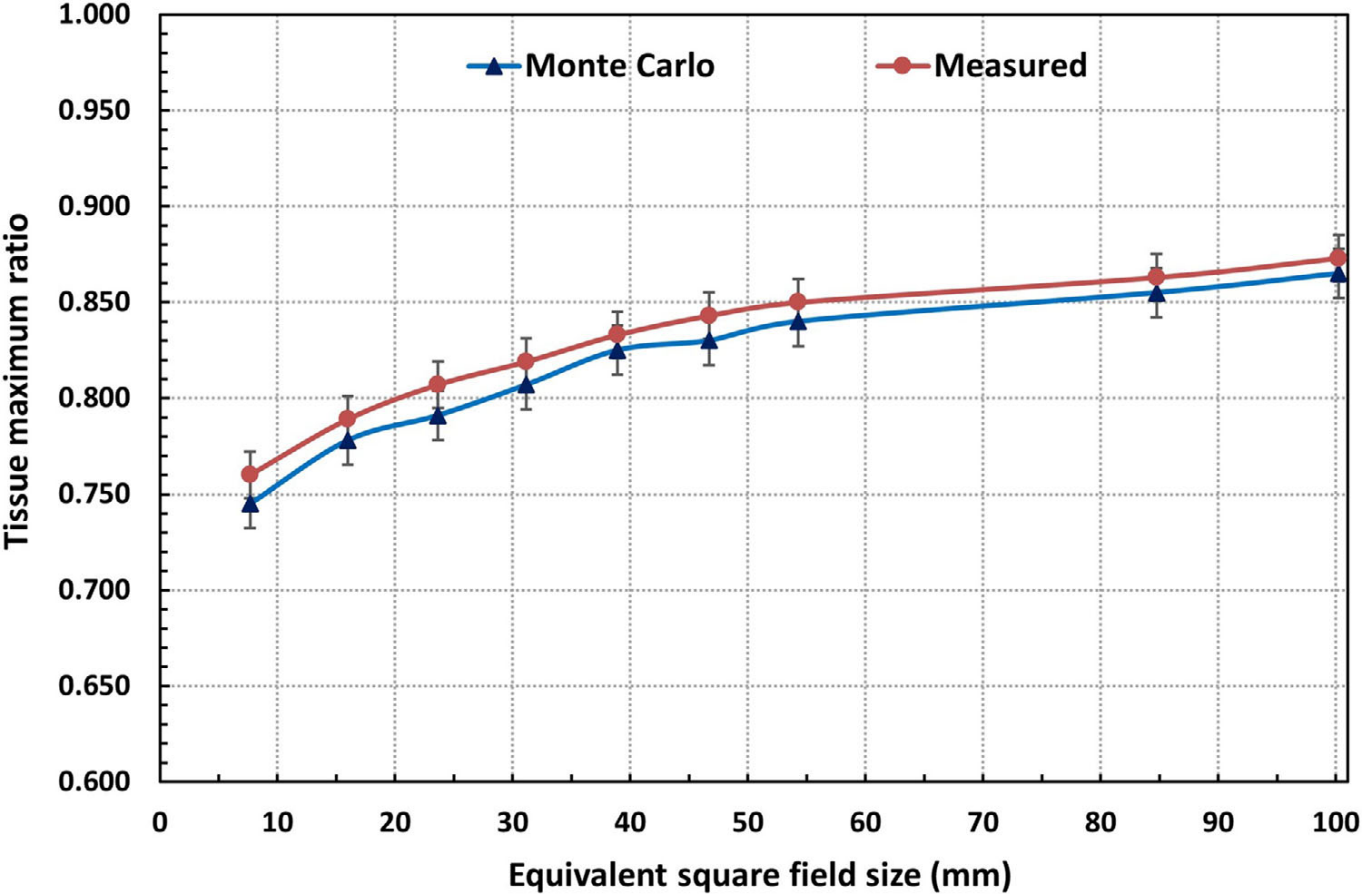
Comparison of tissue maximum ratio (TMR) between the Monte Carlo simulations and water phantom measurements (commissioning data) for static MLC fields of CKM6 system

In Figure 9 the comparison of TMR values between the MC and measurements (commissioned data) for (100.0 × 100.1–7.6 × 7.7) mm^2^ MLC fields at 800 mm SAD, are presented. The average difference between the two was found ≤2%, except for the smallest field tested, for which the % difference rose to 3.5%. It is also noted that the MC simulated TMR values are consistently lower that the measured ones, which could be mainly due to ^1^ the insertion of inaccurate geometry in simulations (difference between the real and simulated geometries), and ^2^ the adoption of different dose calculation modalities in MC (volume‐ based dose calculation) versus TPS (point‐based dose calculation).^11^, ^13^ In the volume‐based dose calculations, the dose is calculated in the voxalized volume leading to volume averaging effect, whereas the point with the maximum dose is considered in point‐based calculations or measurements.

To clarify, the MC‐calculated PDDs or TMRs (for listed static MLC fields) were compared with those obtained from the commissioning measurements, as illustrated in Figure 9. Figures 7 and 8 show the MC simulated depth dose curves for two cases: Water phantom measurements and MapCHECK2 PSQA measurements.

**FIGURE 10:**
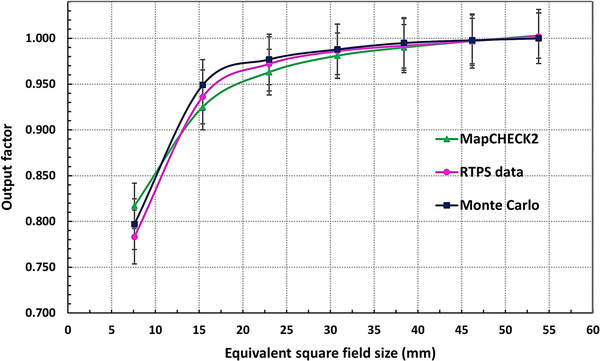
Comparison of output factors (OF) between the Monte Carlo simulations and MapCHECK2 measurements against the RTPS data for (53.8 × 53.9–7.6 × 7.7) mm^2^ MLC fields at 800 mm SAD. The output factors are normalized to the 53.8 mm × 53.9 mm MLC field

In Figure 10 the comparison of normalized OFs between the MC MapCHECK2 measurements and the RTPS data for 53.8 × 53.9–7.6 × 7.7 mm^2^ MLC fields at 800 mm SAD can be seen. The MC simulated OFs differ by 1%, 1.5%, and 2.5% from the RTPS results, for MLC fields ≥23.0 mm × 23.1 mm, 15.4 mm × 15.4 mm, and 7.6 mm × 7.7 mm, respectively. Similarly, the comparison of OFs between the MC simulations OFs and MapCHECK2 measurements showed a ≤2.5% difference. However, the uncertainties of output factors were up to 4.5% for the MapCHECK2 measurements compared with the RTPS data. It is worth to point that since our goal was to investigate the potential field‐specific corrections for the MapCHECK2 (on the CKM6) that can be applied in PSQA, the measured values were not corrected by tabulated values.

**FIGURE 11:**
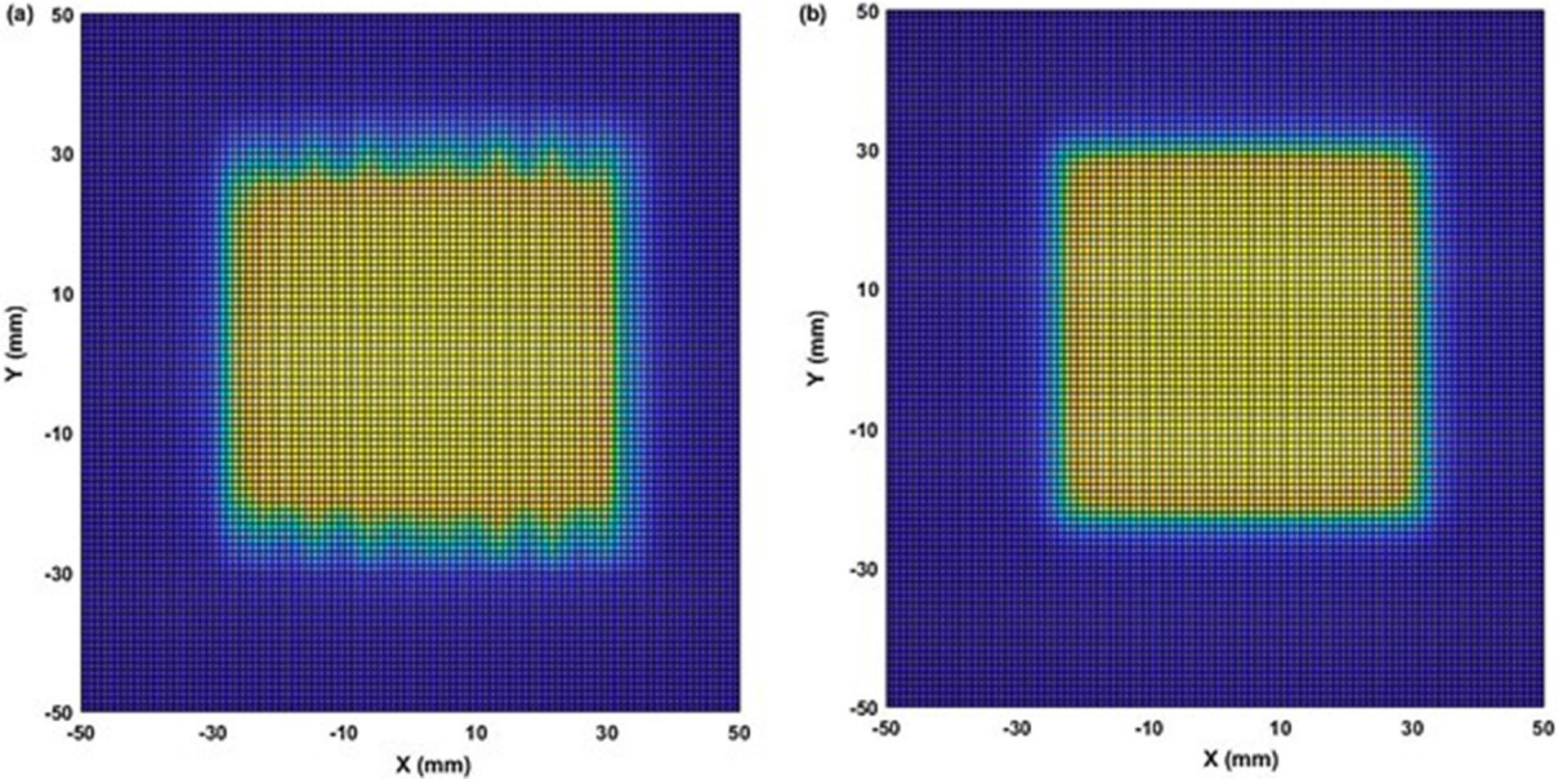
Comparison of planar dose maps obtained from (a) MC simulation and (b) TPS using MapCHECK QA setup (SSD = 730 mm, z = 70 mm) for the MLC field of 53.8 mm × 53.9 mm with 1 mm voxel size in a water phantom

Furthermore, the gamma analysis between the MC simulated and TPS‐generated dose profiles have shown a passing rate of ≥95% with 3%/3 mm and 2%/2 mm gamma criteria. Figure 11 shows planar dose maps generated from the MC simulations versus TPS, for the MLC field of 53.8 mm × 53.9 mm scored at z = 70 mm and SSD = 730 mm (or 800 mm SAD). The MC simulated dose maps (Figure 11a) present a more realistic dose distribution by including fuzzy factors (blurred image at edges due to MLC scatter) that account for complexities of photon attenuations, scatters, and leakage from the MLC leaves Additionally, preliminary results of the phantom study (n = 5) for the PSQA application have shown about a –5% dose difference, based on the 50% isodose distribution between the MC and TPS. The overall difference in the 50%–90% dose range between the TPS and MC was found about 5%–10% in all test cases, which can be used as preliminary or additional PSQA.

### 3.2 Retrospective study

Table 1 shows major clinical parameters recorded from the 50 treatment plans with different beam projections such as anterior‐posterior (AP) and right‐left (RL). An overall gamma passing rate ≥95% was found with 2%/2 mm and 2%/1.5 mm criteria. For 28 treatment plans with segment width > 10 mm, the gamma passing rate was (98.5 ± 1.5)% with AP beam projection, and for six plans with RL beam projection, it was (95.3 ± 2.3)%. However, for 16 highly intensity modulated plans with smaller MLC segments (width ≤ 10 mm), it was (98.0 ± 2.0)% with 2%/1.5 mm criterion after applying approximately ‐ 4% dose correction. The correction factor was applied based on the preliminary results of retrospective measurements for smaller fields. In our opinion, for the smallest field range (∼ 8 mm width for CKM6) 4% correction is within acceptable limits. Several publications reported the correction of up to 5% (for small field diode) for the smallest field.^17^, ^28^

**TABLE 1:** Clinical parameters recorded during retrospective measurements of 50 SRS/SBRT patient plans using the MapCHECK2

Segment width	10–30 mm	10–30 mm	≤10 mm
Beam projection	AP	RL	AP
Gamma index	2%/1.5 mm	2%/1.5 mm	2%/1.5 mm
Passing rate (%)	98.5 ± 1.5	95.3 ± 2.3	98.0 ± 2.0

In addition, the average uncertainty in the measurements was found approximately 3.0% with a 2%/1.5 gamma criterion. Since the application of the measurement uncertainty correction function provided in the patient software tools (SNC) can significantly (9%–14%) inflate the passing rate, thereby masking errors in treatment planning and dose delivery.^35^ Therefore, MapCHECK2 users should not be relying on the fact that a higher passing rate always indicates higher accuracy. It should be noted that even identical measurements may produce significantly different passing rates depending on the specifics of the vendor's algorithms. As we were aware of artificially boosted passing rates with the application of the calculated shift, we noticed a 3%–4% change in the passing rates with or without applying it. Since this function can change the passing rate in the calculated dose distributions rather than in the measured ones and optimize the alignment for better matching of dose maps. The SNC software has a unique tool called “calc shift” or “measurement uncertainty function” that allows one to check the uncertainty in the measurements and how the gamma distance to agreement (DTA) passing rate changes with or without using it. The uncertainty in measurements specifically in very small fields can only be minimized with extremely careful measurements and cross‐examination.^36^

### 3.3 Output factors

**FIGURE 12:**
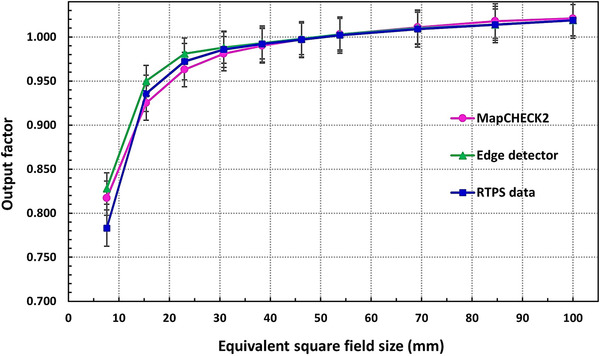
The output factors measured by MapCHECK2 and Edge detectors with 100 MU at 800 mm SAD compared to Radiation Treatment Planning System (RTPS) versus MLC field size. The MapCHECK2 measurements were performed at a 20 mm depth of solid water, while the Edge detector was placed at a 15 mm depth in a water phantom

Figure 12 shows the output factors obtained from measurements using MapCHECK2 and Edge detectors as a function of the field size (i.e., equivalent square field size). The MapCHECK2 measurements compared to the RTPS data have shown very accurate dose results (±0.5% difference) for fields > 30.8 × 30.8 mm^2^, and up to 1.0% under‐response for (30.8 × 30.8–23.0 × 23.1) mm^2^ fields, respectively. However, for the two smallest fields 15.4 × 15.4 and 7.6 × 7.7 mm^2^ the dose under‐response of 1.5% and over‐response of up to 4.5% were observed, respectively. While the Edge detector has shown 2.2% and 5.5% dose over‐responses respectively for these small fields. The results are consistent with previous studies that larger corrections are expected for diodes in smaller fields.^4^, ^17^, ^32^, ^36^

**FIGURE 13:**
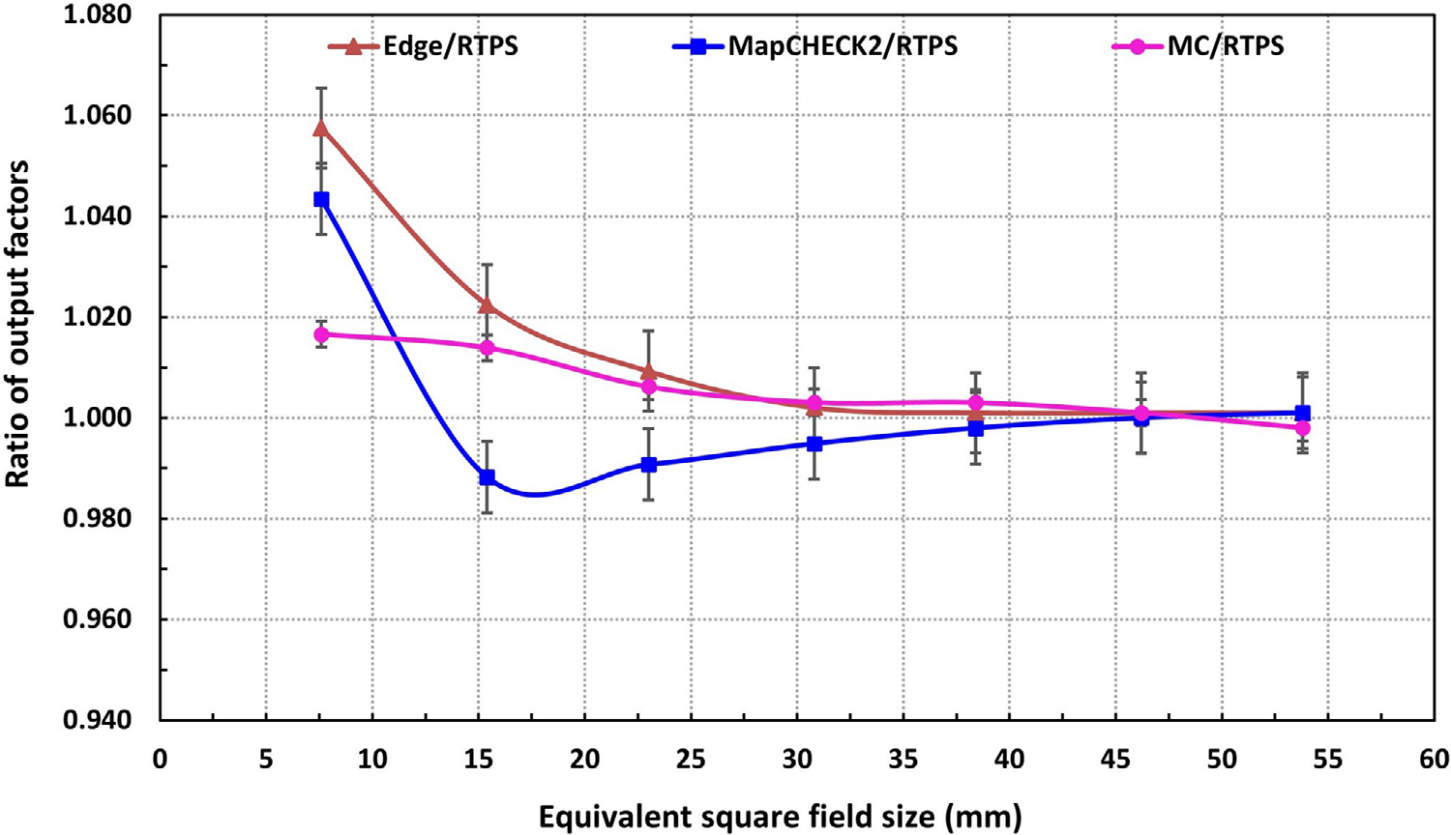
The ratio of the output factors measured by MapCHECK2, Edge detectors and calculated by MC simulations to those from the Radiation Treatment Planning System (RTPS) for (53.8 × 53.9–7.6 × 7.7) mm^2^ MLC fields at 800 mm SAD

In Figure 13, the ratios of the output factors from measurements (MapCHECK2, Edge), and MC simulations to the RTPS data are plotted as a function of field size. The MapCHECK2 outputs exhibited correction ranging from –1.5% (under‐response) to +4.5% (over‐response) in small fields. We observe that the MC calculated outputs stay in between within ± 2.5% uncertainty. These results justify the importance of having a reliable MC model for cross‐examination specifically for small fields. Since a single detector to ensure the accurate dose (±2% uncertainty) for smaller fields is not available,^4^, ^36^ it is recommended to perform very careful measurements including multiple small field detectors as well as apply proper or published correction factors during treatment planning to achieve higher dosimetric accuracy.^8^, ^9^

Our MC dose model utilizes the built‐in graphical user interface (GUI) that makes the MC simulations of static or dynamic fields straightforward. It is the first independent MC dose model reported specifically for the CKM6 radiosurgery system that expands to small field sizes, whose results are compared with the MapCHECK2 PSQA application. Our MC dose model could be further verified by using additional micro‐detectors, SRS MapCHECK2, and/or EBT films,^17^, ^30^ especially for measuring the dose profiles at the tails and shoulders regions as in ref.^49^ In addition, this MC model was created and verified for homogenous water phantom. Further work would be to add CT‐based MC dose calculations, using the ctcreate tool in the DOSXYZnrc dose calculation EGSnrc module, allowing simulations in a realistic anthropomorphic phantom.

## 4 CONCLUSIONS

Overall, an independent MC dose verification model for the CKM6 radiosurgery system was developed and validated for static MLC fields with good agreement (≤3% difference) for all fields, but a 4.5% difference for the smallest field. The measurements of MLC plans with MapCHECK2 have shown variable dose responses in smaller fields with a deviation from –1.5% to +4.5% compared to the RTPS results, while the MC simulated dose outputs fall within ± 2.5% tolerable uncertainty. Even though the dose variations between the TPS data, MapCheck2 measurements, and water phantom measurements, all corresponding PSQAs performed passed the applied clinical acceptance of treatment plans. The current results demonstrate the feasibility and efficacy of the presented MC dose model for patient‐specific QA as a supplemental dose verification tool.

## AUTHOR CONTRIBUTIONS

Conceptualization, Taindra Neupane, Charles Shang, and Wazir Muhammad; methodology, Wazir Muhammad; validation, Charles Shang, and Michael Kasper; formal analysis, Taindra Neupane, Panagiota Galanakou, Wazir Muhammad, and Theodora Leventouri; investigation, Taindra Neupane, Panagiota Galanakou, Wazir Muhammad, and Charles Shang; Original draft preparation, Taindra Neupane; writing—review and editing, Wazir Muhammad, Panagiota Galanakou, Theodora Leventouri, Charles Shang, and Michael Kasper; supervision, Wazir Muhammad, and Charles Shang.

## CONFLICT OF INTEREST

All authors have read and agreed to the published version of the manuscript.
